# Nanopore base calling on the edge

**DOI:** 10.1093/bioinformatics/btab528

**Published:** 2021-07-27

**Authors:** Peter Perešíni, Vladimír Boža, Broňa Brejová, Tomáš Vinař

**Affiliations:** Department of Applied Informatics, Faculty of Mathematics, Physics and Informatics, Comenius University in Bratislava, Mlynska dolina, 842 48 Bratislava, Slovakia; Department of Applied Informatics, Faculty of Mathematics, Physics and Informatics, Comenius University in Bratislava, Mlynska dolina, 842 48 Bratislava, Slovakia; Department of Computer Science, Faculty of Mathematics, Physics and Informatics, Comenius University in Bratislava, Mlynska dolina, 842 48 Bratislava, Slovakia; Department of Applied Informatics, Faculty of Mathematics, Physics and Informatics, Comenius University in Bratislava, Mlynska dolina, 842 48 Bratislava, Slovakia

## Abstract

**Motivation:**

MinION is a portable nanopore sequencing device that can be easily operated in the field with features including monitoring of run progress and selective sequencing. To fully exploit these features, real-time base calling is required. Up to date, this has only been achieved at the cost of high computing requirements that pose limitations in terms of hardware availability in common laptops and energy consumption.

**Results:**

We developed a new base caller DeepNano-coral for nanopore sequencing, which is optimized to run on the Coral Edge Tensor Processing Unit, a small USB-attached hardware accelerator. To achieve this goal, we have designed new versions of two key components used in convolutional neural networks for speech recognition and base calling. In our components, we propose a new way of factorization of a full convolution into smaller operations, which decreases memory access operations, memory access being a bottleneck on this device. DeepNano-coral achieves real-time base calling during sequencing with the accuracy slightly better than the fast mode of the Guppy base caller and is extremely energy efficient, using only 10 W of power.

**Availability and implementation:**

https://github.com/fmfi-compbio/coral-basecaller

**Supplementary information:**

Supplementary data are available at *Bioinformatics* online.

## 1 Introduction

MinION by Oxford Nanopore Technologies (ONT) is a portable DNA sequencer that measures electric current as DNA passes through nanopores. Electrical signals produced by the device need to be translated into sequences by a base caller software. In many applications of nanopore sequencing, fast and accurate base calling has become a major bottleneck. In bioinformatics, it has been previously demonstrated that such bottlenecks can be solved not only by algorithmic changes but also by using more suitable hardware architectures. These include both readily available consumer devices such as GPUs, but also devices that are the result of hardware-software codesign (Alser *et al.*, 2021; Cali *et al.*, 2020; Fujiki *et al.*, 2018; Kim *et al.*, 2018; Turakhia *et al.*, 2018). In fact, most of the existing tools available for nanopore base calling require powerful GPUs with high energy consumption to operate at reasonable speeds (Oxford Nanopore Technologies, 2020; Teng *et al.*, 2018).

In this paper, we present a new base caller DeepNano-coral, which runs on the Coral accelerator featuring the Edge tensor processing unit (TPU), a small, energy-efficient and cheap USB-connected device. DeepNano-coral can process approximately 1.5 million signals per second, which is enough to provide real-time base calling for a MinION device. The main motivation behind using this particular device is a practical applicability of our solution: Coral is a readily available consumer device, which is easily connected to a common laptop computers. This makes our base caller ideal for field sequencing applications, where power efficiency and low hardware requirements are highly desirable. Real-time base calling is also essential in unlocking some of the most promising MinION device capabilities, such as its ability to adapt the run length to the sample composition, or selective sequencing (Loose *et al.*, 2016).

Current base callers are typically based on deep neural networks. Guppy, a base caller provided by ONT, is based on recurrent neural networks (RNN) and provides two different architectures: a fast base caller, which can base call with 85–92% median read accuracy in real time, using recent GPU cards and a high-accuracy base caller (90–96% median read accuracy), which is too slow to be used in real time without specialized setup. DeepNano-blitz trades off a bit of accuracy in order to provide real-time base calling on a common CPU using a specifically engineered RNN, thus obviating the need for GPUs (Boza *et al.*, 2020). Other RNN-based base callers, including Chiron (Teng *et al.*, 2018), are too slow for real-time base calling. Another class of nanopore base callers is based on convolutional neural networks (CNN). In particular, Bonito v.0.2 (Oxford Nanopore Technologies, 2020) adapts Jasper/QuartzNet (Kriman *et al.*, 2020; Li *et al.*, 2019) speech recognition architecture to base calling tasks. At the time of writing, Bonito provided the most accurate base calling, however, the time requirements exceed even the Guppy high-accuracy mode.

The Coral Edge TPU accelerator by Google is a limited device, which was designed mostly for vision tasks, such as image classification (Google Research, 2020). It contains only 8 MB of memory (used for storing both model weights and intermediate tensors), it only works with 8-bit integers (while GPUs typically work with 32-bit floating point numbers) and the compiler and libraries provide only a limited set of building blocks, optimized mostly for CNNs with small receptive fields, which are typically used in image processing. Such a configuration mostly excludes possibility of adaptation of RNN-based architectures and even adapting CNN-based architectures, such as Bonito, is a challenge, due to the large size of the network and the use of large receptive fields.

We show that by introducing several novel features in the CNN-based base calling architecture, one can overcome these limitations of the Coral devices. Our new base caller DeepNano-coral provides real-time base calling that is significantly more energy efficient than existing approaches running on GPU (e.g. Guppy base caller) or CPU (Boza *et al.*, 2020). These advantages, coupled with easy availability and a low price of Coral devices, make this a practical solution for the problem of real-time base calling.

To achieve this goal, we introduce the following innovations:

- A novel component ***k*-blueprint-separable-convolution**, which replaces separable convolutions as a building block for CNNs. A separable convolution approximates a full convolution by using a depthwise operation and a pointwise operation, which are less computationally intensive. The *k*-blueprint-separable convolution factorizes the convolution into the two parts differently, in effect reducing the depthwise operation at the cost of increasing computation in the pointwise operation. Even though the new convolution component has a higher number of parameters and floating point operations (flops), it is more efficient on the Edge TPU, since in this architecture, depthwise operations do not fully utilize the hardware (Gupta and Akin, 2020; Xiong *et al.*, 2021), possibly due to being bound by the memory bandwidth.
- **A new design of the residual block**, which is a fundamental building block of the QuartzNet speech recognition architecture and was also deployed for base calling in Bonito. To improve its performance on the Edge TPU, we add a compression operation at the start of the residual block, taking *x* consecutive data samples of *C* channels each and converting them into a single compressed sample of *Cy* channels. Using compression ratio x/y<1, we save memory and allow subsequent convolutions to effectively mix *x* original samples and thus increase the receptive field of the block.
- A surprising observation that **identity initialization of some parts of the architecture helps the training and improves the prediction accuracy** in some circumstances, which contradicts usual recommendations for initializing parameters of neural networks before training.

Our experiments show that DeepNano-coral achieves the accuracy comparable to other real-time base callers, Guppy fast and DeepNano-blitz. Such accuracy is sufficient for real-time monitoring tasks, such as monitoring barcode composition in pooled libraries or species composition in environmental or clinical samples (Boza *et al.*, 2020). DeepNano-coral achieves this goal much more energy efficiently, using only 0.6–0.7 Wh of energy to base call a test sample of 40.8 Mb of nanopore sequences at a speed of 1.54 million signal samples per second (on the same setup, the closest competitor, Guppy fast, uses 1.4 Wh of energy, processes 4.37 million signal samples per second, with up to 2 percentage points lower accuracy depending on the dataset).

*Background*. Nanopore base calling translates the electrical signals produced by the sequencer into a sequence of DNA bases. The signal level depends on the context of about 5–12 DNA bases passing through the nanopore. The signal is read about 4000 times per second and DNA moves through the pore at the speed of ∼450 bases per second, but the speed is rather uneven. This means that on average each shift of the context by one base corresponds to roughly 9 measured values with a large variance. This makes the problem somewhat similar to speech recognition. Note that different contexts may produce very similar signal levels and that there is a significant amount of noise present in the signal readouts, complicating the base calling problem.

To address complex interactions of the context and noise in the signal readouts, current state-of-the-art base callers use neural networks to predict DNA bases passing through the pore. While RNNs propagate state information as they process sliding windows of the input signal, CNNs process an entire fixed-sized window of the signal in parallel (see Goodfellow *et al.*, (2016) for an overview of CNNs and RNNs). In this work, we use the CNN architecture.

A convolutional layer is the dominant basic building block of many neural network architectures, mostly in the domain of image recognition, but recently also for automated speech recognition (Kriman *et al.*, 2020; Li *et al.*, 2019; van den Oord *et al.*, 2016). In this paper, we consider 1 D convolutions, which take as an input a tensor of dimensions $\left(T,C_{\text{in}}\right)$ representing a data stream of length *T*, each data point containing *C*_in_ values called *channels*. They produce an output tensor of dimensions $\left(T,C_{\text{out}}\right)$ by applying a linear transformation to sliding windows of size *D* (*convolution depth*) of the input tensor (see details in Section 2).

Through the years, the deep learning community went from networks with simple convolutional layers to more complex layers which use skip connections and specific special classes of linear transformations. These changes led to improvements in training and inference speed as well as in training convergence. The layers are typically organized in a fractal design, where the architecture is composed of several high-level layers, and these are in turn composed of more basic computation units. We depict both simple and more complex layers by rectangles and indicate the use of results from one layer in another layer by arrows.

**Fig. 1.**
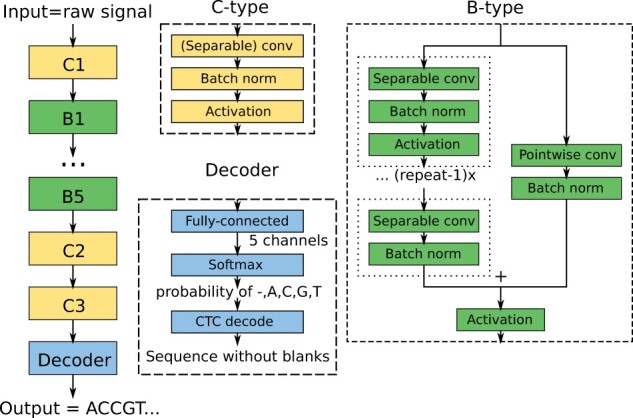
Bonito CNN-based architecture. The architecture is composed of high-level blocks depicted as colored rectangles, where the output of the previous block serves as an input of the following block. The neural network is composed of three blocks of type C, five blocks of type B and a Decoder block. The construction of these block types is depicted on the right; each block type is composed of standard building blocks used in deep learning

The work in this paper is based on the QuartzNet architecture (Kriman *et al.*, 2020) for speech recognition, which has also been used in the Bonito base caller (Oxford Nanopore Technologies, 2020) developed by ONT. Briefly, a window of the raw signal of length *T* is used as an input to a deep CNN which uses several types of blocks to process the signal (Fig. 1). In the final decoder block, the network produces a tensor with five output channels. For each output position, the values of the five channels are converted by the softmax function into a probability distribution over possible outputs A, C, G, T,-, with dash corresponding to an empty output. Finally, the CTC layer (Graves *et al.*, 2006) chooses a DNA sequence with the highest posterior probability.

In the QuartzNet/Bonito architecture, convolutions are organized into building blocks of two types B and C. The structure of a C-type block is simply a sequence of three layers: a convolutional layer, a batch normalization (Ioffe and Szegedy, 2015) (a layer that renormalizes channel values and stabilizes gradients for better training) and an activation function [Bonito uses Swish (Ramachandran *et al.*, 2018)].

The B-type blocks use residual skip connections. The input signal is split into two branches. The main branch consists of *R* copies of a C-type sub-block, with the last copy omitting the activation function. The second branch, called skip connection, consists of a pointwise convolution and batch normalization. The two branches are summed together and an activation is applied to the output.

The resulting network used in Bonito is large and computationally intensive. Some intermediate results reach size of up to B×T/3×464, where *B* is the number of sequences combined to a batch and *T* is the length of the sequence. The network has 36 convolutional layers with 6.6 million parameters in total, requiring roughly 2.2 million multiplications per sample.

## 2 Materials and methods

In this section, we present the architecture of our new base caller designed for the Edge TPU. Our architecture is inspired by the Bonito CNN, which was drastically scaled down and key components were replaced by the enhancements described here. Further technical details regarding adapting Bonito-like architecture to the Edge TPU are described in the Supplementary Material.

*The k-blueprint-separable convolutions*. The basic form of a convolutional layer is a *full convolution*, which takes as an input a tensor *X* of dimensions $\left(T,C_{\text{in}}\right)$ and produces an output tensor *Y* with dimensions $\left(T,\;C_{\text{out}}\right)$. To apply a convolution with odd depth *D*, the input tensor *X* is first padded with $\left\lfloor \frac{D}{2}\right\rfloor$ zeros at the beginning and at the end. The output is then computed as follows: $Y_{t,j}=\sum_{0\leq d<D,0\leq i<C_{\text{in}}}X_{t+d,i}W_{j,d,i}+B_{j},$ where *W* and *B* are trained weights representing convolution kernel weights and bias, respectively.

**Fig. 2.**
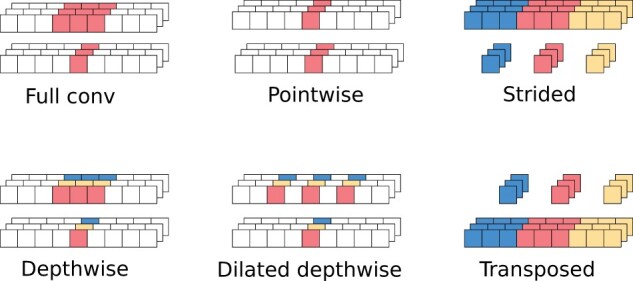
Receptive fields for basic types of convolutions. For each convolution type, the two rows represent input and output data of the convolution. Multiple channels are stacked. The colored value in the output tensor is computed from the values of the same color in the input tensor

An obvious drawback of full convolutions is a large number of parameters ($C_{\text{out}}DC_{\text{in}}$) and required flops ($TC_{\text{out}}DC_{\text{in}}$). A standard solution is to use a *separable convolution* (Mamalet and Garcia, 2012), which is an approximation of the full convolution by a composition of two operations: depthwise and pointwise. The *depthwise operation* works on each channel separately: $Z_{t,j}=\sum_{0\leq d<D}X_{t+d,j}W_{d,j}^{\left(D\right)}+B_{j}^{\left(D\right)}$. This is followed by the *pointwise operation*, which mixes the channels at each time point: $Y_{t,j}=\sum_{0\leq i<C_{\text{in}}}Z_{t,i}W_{j,i}^{\left(P\right)}+B_{j}^{\left(P\right)}$. This reduces the flops from $TC_{\text{out}}DC_{\text{in}}$ to $T(DC_{\text{in}}+C_{\text{out}}C_{\text{in}})$. The ordering of pointwise and depthwise operations was chosen somewhat arbitrarily, and reversing it may improve the accuracy (Haase and Amthor, 2020). The variant with the reversed order is called a *blueprint-separable convolution*. Figure 2 illustrates receptive fields of basic operations used in convolutions.

Recent works (Gupta and Akin, 2020; Liu, 2020; Xiong *et al.*, 2021) indicate that separable convolutions do not always improve the speed on non-CPU architectures, because the depthwise operation requires a smaller ratio of flops to memory operations, which are generally slow. A full convolution with depth *D *=* *3 can be faster than a separable convolution with the same depth (Xiong *et al.*, 2021). Full convolutions with small depths are thus feasible in image recognition, while in base calling, the kernels need to be much larger.

**Fig. 3.**
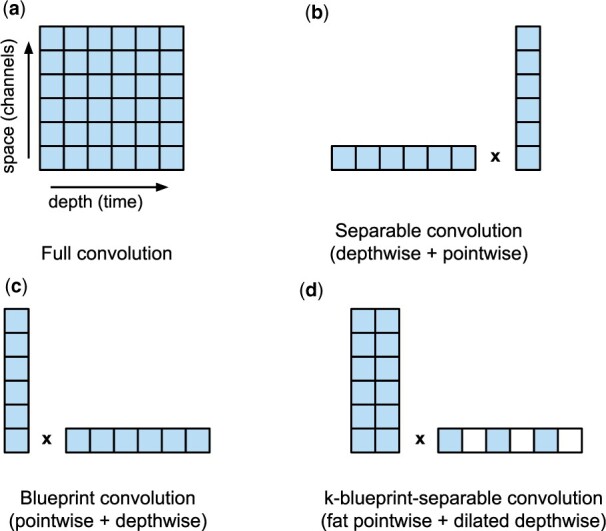
Comparison of different convolution factorizations. Individual filled squares represent input values convolved with a weight tensor to a single output or intermediate value. Our *k*-blueprint-separable convolutions introduce a novel combination of basic blocks. (**a**) $C_{\text{in}}=C_{\text{out}}=128$ and tensor size (4, 1668, 128) and (**b**) $C_{\text{in}}=C_{\text{out}}=256$ and tensor size (4, 556, 256)

Our design of *k*-separable convolutions is heavily influenced by this observation. Our goal is to reduce the time-consuming depthwise operations using dilation with step size *k*, and compensate by replacing the pointwise operation by a convolution operating on a window of small size *k* instead of a single point, as illustrated in Figure 3.

Namely, we start with what we call a *fat-pointwise* operation, which is a standard convolution of depth *k*:

$$Z_{t,j}=\sum_{0\leq d<k,0\leq i<C_{\text{in}}}X_{t+d,i}W_{j,d,i}^{\left(P\right)}+B_{j}^{\left(P\right)}.$$

The second step uses a dilated depthwise operation with depth *D*/*k*, which skips points by using dilation *k*: $Y_{t,j}=\sum_{0\leq d<D/k}Z_{t+dk,j}W_{d,j}^{\left(D\right)}+B_{j}^{\left(D\right)}$. This reduces the depthwise kernel (and thus memory I/O) by a factor of *k*, while retaining the receptive field *D* of the whole layer.

Note that the special case of *k *=* *1 leads to a standard blueprint convolution, while we typically use *k *=* *3, which on the Edge TPU roughly maintains the same computation time as separable convolutions, while increasing the accuracy.

**Fig. 4.**
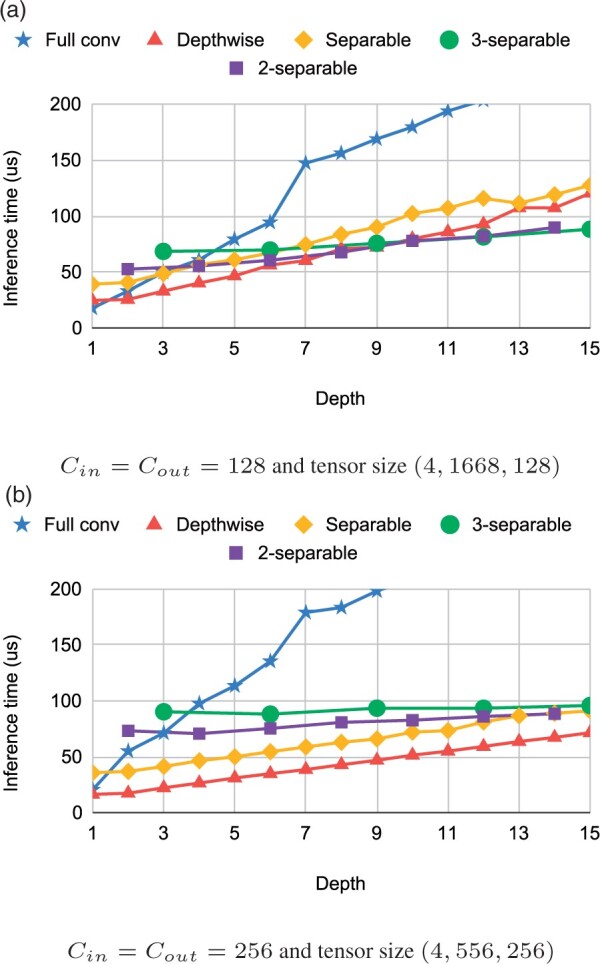
Inference time of different convolutions on the Coral device. Note that pointwise corresponds to a full convolution with depth = 1

Figure 4 demonstrates the performance of *k*-separable convolutions on two configurations used in our experiments in the next section. Our *k*-separable convolutions offer running times comparable to separable convolutions, while providing roughly *k* times more parameters, which increases their expressive power.

**Fig. 5.**
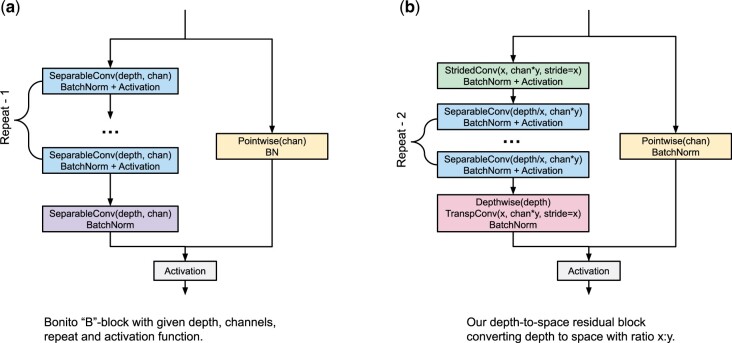
Residual block with depth-to-space compression (right) is a drop-in replacement for a regular Bonito B-type block (left). The first separable convolution is replaced by a compression block, which compresses the input tensor dimensions from (*T*, *C*) to (T/x,Cy). Subsequent separable convolutions use depth reduced by a factor of *x* and channels increased by a factor of *y*. The final separable convolution is replaced by a decompression block

*Residual block with depth-to-space compression*. Our second change also targets reduction of the depthwise convolution. As shown in Figure 4, when we apply convolutions on a shorter input, we can use more channels in a comparable time. Our idea is to redesign the residual block of the CNN (B-type block in Fig. 1) so that we compress its depth and increase the number of channels. In particular, compression with depth-to-space ratio x:y means converting input tensor (*T*, *C*) to tensor (T/x,Cy) using a strided convolution with both depth and stride set to *x* (Fig. 2). This convolution takes *x* consecutive data samples of *C* channels and converts them into a single compressed sample of *Cy* channels. At the end of the residual block, we restore the original dimensions with a strided transposed convolution. This makes the new block a drop-in replacement for the original B-type block design (Fig. 5).

Compression ratio x/y<1 saves memory, which is essential due to limited Coral resources. While compression may sometimes decrease accuracy, the network may learn to de-duplicate information from consecutive data samples, and thus prevent data loss. In fact, any subsequent pointwise operations effectively operate on *x* original samples, yielding increased receptive fields. Thus, we can further lower the depth of the depthwise operation in the block, offsetting larger computation of pointwise operations, which were increased by a factor of $y^{2}/x$. In our experiments, compression ratio 3:2 works well on Coral.

To complete the residual block, we add the depthwise operation before the decompression. While the original B-type block repeats separable convolutions *R* times, we repeat them *R—*2 times, since we consider the compression and decompression blocks as replacements for two separable convolutions.

*Identity initialization*. A proper neural network initialization can affect both trainability and final accuracy of models (Glorot and Bengio, 2010; Le *et al.*, 2015; Mishkin and Matas, 2016; Sutskever *et al.*, 2013; Zhang *et al.*, 2019). A standard way of initializing CNN architectures is to draw the entries of weight matrices from the uniform distribution U(−k,k), where $k=6/\sqrt{C_{\text{in}}+C_{\text{out}}}$, and to set the bias terms to zero (Glorot and Bengio, 2010). The weighting factor *k* is used to keep the gradients from vanishing or exploding as the number of layers increases. Recent introduction of BatchNorm however obviates such problems, as the results are renormalized (Ioffe and Szegedy, 2015).

In some cases, task-specific initialization may bring an improvement over the generic initialization strategies (Le *et al.*, 2015), and this proved to be the case for our base calling application as well. We initialize all *k*-separable blocks within the compressed main branch to near-identity, that is, depthwise kernels are initialized as $W_{d,j}^{\left(D\right)}=\delta_{\left\lfloor (D/K)/2\right\rfloor ,d}$ and fat pointwise kernels to $W_{j,d,i}^{\left(P\right)}∼\delta_{\left\lfloor K/2\right\rfloor ,d}(\delta_{i,j}+U(-\epsilon ,\epsilon )),$ where $\delta_{x,y}=1$ if and only if *x *=* y*. We experimented with several other initializations and observed that setting the depthwise operations to identity helps the most, while setting pointwise operations to identity brings only a small additional improvement. On the other hand, initialization of the skip connection as well as of the compression/decompression block does not seem to affect the results significantly.

In our experiments, the identity initialization described above speeds up the process of training and decreases overfitting (Section 3). We believe that this surprising effect is explained by the properties of the base calling task.

Due to the nature of nanopore raw sequencing data, base calling is composed of two tasks. First, the input signal needs to be segmented into events, each event corresponding to the shift of the DNA currently read by the nanopore head by one base. The length distribution of these events is highly variable. The second task is to recognize the base under the nanopore head given the context of several events. Although initially base callers have performed these tasks separately, modern neural network approaches combine them into a single optimization problem.

One would assume that the second task of correctly identifying bases is the core of the problem. A quick experiment in which Bonito is provided with an additional binary input indicating event boundaries (as determined from a ground-truth alignment to a reference) shows otherwise. In particular, the additional input dramatically speeds up the training so that the network can in minutes outperform days-long Bonito training. While the modified network cannot be used for practical base calling (because the base caller obviously cannot receive ground-truth event boundaries as an input), it suggests that identification of events is in fact the harder part of the base calling task. This is further corroborated by the fact that even a simple logistic regression can distinguish purines A, G from pyrimidines C, T in a correctly segmented signal.

Our depthwise identity initialization indeed makes sense assuming that the network spends much more time learning how to split the raw signal into events rather than recognizing individual bases. Identity initialization may allow the network to learn the easy task of distinguishing bases first and then spend the rest of its capacity on learning intricate time-dependencies without the need for unlearning spurious long-range correlations that may have been introduced by random initial weights.

## 3 Results

In this section, we compare the speed, energy consumption and accuracy of DeepNano-coral with other tools on a dataset of R9.4.1 reads from *Klebsiella pneumoniae* (Wick *et al.*, 2019) and human (Jain *et al.*, 2018) (Supplementary Material). The base calls were mapped to the reference using minimap2 (Li, 2016).

DeepNano-coral slightly outperforms Guppy fast in most accuracy measures (Table 1). Guppy fast would currently be a method of choice for live base calling on a computer with a recent GPU card (compute capability 6.2, 4 GB of memory). As demonstrated earlier (Boza *et al.*, 2020), even slightly lower accuracy of DeepNano-blitz is sufficient for run monitoring, such as barcode composition or metagenomic analysis. Note that DeepNano-blitz provides real-time base calling on a CPU without the use of any accelerator. Guppy in the high accuracy (hac) mode illustrates accuracy gains possible with more extensive computational resources typically beyond the possibilities of real-time base calling.

**Table 1.** Comparison of base calling accuracy

	*K.pneumoniae*		Human	
Base caller	Mapped (%)	Median accuracy (%)	Mapped (%)	Median accuracy (%)
Guppy 4.0.11 hac	100	94.7	93.4	89.8
Guppy 4.0.11 fast	100	91.2	92.1	84.7
DeepNano-coral	100	92.4	88.5	87.2
DeepNano-blitz 80	100	90.4	86.1	84.3

*Note*: Read accuracy is computed as one minus the ratio of the alignment edit distance and the base call length. We report the median read accuracy.

We have measured the speed and energy consumption on two computers with different setups (Table 2), a desktop (i7-7700k 4 core CPU; NVIDIA GTX 1650 GPU) and a laptop (i7-7700HQ 4 core CPU) incapable of running the GPU version of Guppy. To run DeepNano-coral, we have attached the Coral Edge TPU device through USB 3.0 interface.

**Table 2.** Energy consumption and speed of different base callers (DN, DeepNano)

Base caller	Power (W)	Speed (signals/s)	Time (s)	Energy for base calling (Wh)	Total energy (Wh)
Desktop					
Idle baseline	62	––	––	––	––
DN-coral	72–73	1.52 M	234	0.68	4.71
DN-blitz 80 (4 threads)	168–170	2.12 M	168	4.94	7.84
DN-blitz 80 (2 threads)	120–122	1.13 M	316	5.09	10.53
Guppy fast	110	3.32 M	107	1.42	3.27
Guppy hac	135	79 k	4495	91.14	168.56
DN-coral on GPU	154	1.34 M	265	6.8	11.3
Laptop					
Idle baseline	18	––	––	––	––
DN-coral	27	1.51 M	235	0.58	1.76
DN-blitz 80 (4 threads)	73	1.53 M	232	3.54	4.70
DN-blitz 80 (2 threads)	56	907 k	392	4.13	6.09

On both computers, DeepNano-coral achieved the speed necessary for live base calling (1.5 M signals per second) and used less than 11 W (computed as a difference between the idle energy consumption and the consumption during base calling). On our testing set, the total energy spent on base calling was 0.58–0.68 Wh, roughly half of the energy used by Guppy fast on the desktop. Although Guppy fast consumed less energy when baseline is included due to its shorter running time, in a practical setting, this would not translate to energy savings as the computer needs to run throughout the sequencing.

DeepNano-coral runs on a GPU at lower speed and with higher energy consumption than GPU- and CPU-optimized software. This underlines the importance of optimization of the network architecture for a particular platform.

To further illustrate the impact of our new network designs on the base calling accuracy, we started with the small Bonito architecture (Supplementary Material), in which we replaced various components by our new designs presented in the Section 2. In these experiments, we modify only B-type (residual) blocks, keeping the standalone C-type blocks the same. We however verified that altering configuration of these C-type blocks does not affect the accuracy significantly.

**Fig. 6.**
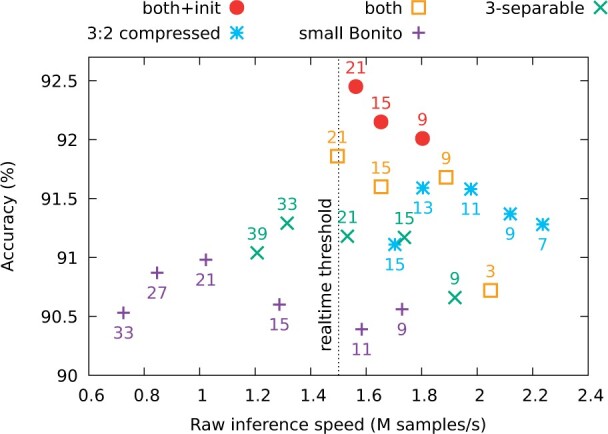
Speed-vers us-accuracy frontier for various architectures and depthwise kernel sizes. Each point represents the tradeoff for a given kernel size

Figure 6 shows the accuracy and speed starting with the small Bonito and adding the following features: 3-blueprint-separable convolutions, compression with ratio 3:2, combination of the two and finally the identity initialization. For each variant, we test several kernel depths. Note that 3-separable convolutions have a symmetrical receptive field only for depth of size k=3(2n+1). In most experiments, we stop at kernel size 21, because larger kernels lead to base calling speed below the speed of sequencing. In general, adding our modifications increases the accuracy at comparable speed, and the most accurate version is the one with all our improvements combined.

## 4 Discussion

In our work, we combine novel improvements to the existing base calling architecture with an emerging off-the-shelf acceleration device in order to solve an important problem of real-time base calling of nanopore sequencing data. In particular, we have designed new types of blocks which can be used as drop-in replacements for separable convolutions and QuartzNet-style residual blocks, potentially improving their speed/accuracy tradeoff in other applications as well.

From a practical standpoint, our work enables real-time base calling with low energy consumption on modest hardware with addition of a $70 USB device. DeepNano-Coral provides a better accuracy than Guppy-fast, which is currently a standard tool for real-time base calling when using GPUs. This contribution will help researchers attempting nanopore sequencing in field conditions with limited energy resources. Using Edge TPU as an alternative to GPU chips may also help to design new devices specifically targeted at nanopore sequencing, analogous to the MK1C device manufactured by ONT.

Further research into decreasing the size of the base calling neural networks may yield even better results on small accelerators. One option is to use knowledge distillation (Hinton *et al.*, 2015), where a smaller network is trained on outputs from a larger network. Another avenue is to consider a richer set of outputs from the network. In our case, the softmax layer output probabilities over the {A,C,G,T,−} alphabet, which is followed by CTC decoding. Guppy and Bonito v0.3 use a more complicated scheme, which could be adapted. The risk here is that we would need to do more intensive decoding on the CPU, which may become a bottleneck.

## Funding

This work was supported in part by grants from the Slovak Research Grant Agency VEGA 1/0458/18 (to T.V.) and 1/0463/20 (to B.B.), by the grant from Slovak Research and Development Agency APVV-18-0239 and by funding from the European Union’s Horizon 2020 research and innovation programme under grant agreement No 872539 (PANGAIA). The work has also been supported by Google Cloud (to V.B.). The authors gratefully acknowledge the support of NVIDIA Corporation with the donation of hardware used in this research.

*Conflict of Interest*: none declared.

## Data availability

No new data were generated or analysed in support of this research.

## Supplementary Material

btab528_Supplementary_Data
