# Supplementary material for: Nanopore base calling on the edge
Source: Bioinformatics. 2021 Jul 27;37(24):4661–7. doi: 10.1093/bioinformatics/btab528 (PMC8665737; doi:10.1093/bioinformatics/btab528)
Supplement: btab528_Supplementary_Data [file btab528_supplementary_data.pdf]

Nanopore Base Calling on the Edge
Supplementary Material

Peter Perešíni, Vladimír Boža, Broňa Brejová, and Tomáš Vinař

Faculty of Mathematics, Physics and Informatics, Comenius University in Bratislava, Mlynská dolina, 842 48 Bratislava, Slovakia

S1 Engineering neural networks for Coral Edge TPU

In this section, we discuss practical steps needed to adapt the Bonito GPU-tuned base caller architecture to run on and fully utilize Edge TPU accelerator. These observations can be useful also to others who want to optimize different neural network architectures for this platform.

Our first step was to create a scaled-down version of Bonito, capable of running on the Edge TPU. Bonito uses large convolutions, with up to 464 output channels. Our experiments suggest that the performance of the Edge TPU accelerator severely deteriorates beyond approximately 128 channels. To stay safely within these bounds, we decrease the maximum number of channels to 128. We also scale down the depth of the depthwise operation, considering the range of 9-33, as the performance cost of larger depthwise kernels is noticeable (see the main text). The final version of DeepNano-coral uses kernel depth 21.

To avoid an extensive architecture search, we use a more uniform configuration of building blocks. In particular, all residual B-type blocks use five convolution blocks, which is a middle ground compared to the original Bonito configuration.

Another issue is related to the quantization. It would be ideal to fuse the activation function to the preceding convolution layer, but the development tool chain does not support this. It also does not support the Swish [Ramachandran et al., 2018] activation function used in Bonito. For these reasons, we have used ReLU6 as the activation function, which can be fused into preceding convolution layers by a simple value clipping of int32 accumulator values used in matrix multiplication.

Further issues are related to limitations of the available development tools for the Edge platform. Typically, neural network inference is done in batches, with several inputs processed simultaneously to optimally utilize hardware capacity. However, this setup is not supported in the current development tool chain. Another problem is that 1D convolutions are internally converted to 2D convolutions, which adds a reshape operation before and after the convolution. This has a severe performance impact, since reshape operations are memory intensive.

To solve both these problems at the same time, we transform multiple 1D inputs into a single 2D "image" of dimensions $B \times T$, where B is the batch size and T is the sequence length. The whole network is then rewritten to an equivalent network operating on this "image" using 2D convolutions. In our network, considering the utilization of the device and the target speed, we use $B = 4$ and $T = 5004$ (T must be a multiple of 9). Note that splitting the input signal into slightly overlapping chunks of 5004 observations is a reasonable compromise between overhead imposed by the overlaps and the capacity of the device.

After these modifications, we obtain a small Bonito-like architecture which the Edge TPU compiler is able to fit on the device. The overall architecture configuration is summarized in Table S1. Finally, the last softmax layer as well as CTC decoding are performed directly on the CPU.

Table S1: Baseline small Bonito architecture we use in our experiments.

C1	Conv(filters=128, depth=9, stride=3) + BatchNorm + ReLU6
B1-B5	Residual with 5x SeparableConv(filters=128, depth= 7-33)
C2	SeparableConv(filters=128, depth=11) + BatchNorm + ReLU6
C3	Conv(filters=64, depth=7) + BatchNorm + ReLU6
Decoder	Pointwise(filters=5) + Softmax

S2 Training and Testing Sets

We have used the combination of the following public data sets to train the models:

- *Taiyaki set:* Data set of 50k (downsampled to 5k) R9.4.1 reads from a PCR amplified DNA of *E. coli* (SCS110), *H. sapiens* (NA12878), and *S. cerevisiae* (NCYC1052), published by Oxford Nanopore as a part of Taiyaki software (https://github.com/nanoporetech/taiyaki/).
- *E. coli set:* A sample of 2804 ultra-long native R9.4.1 reads of *E. coli* (MG1655) from Loman Lab (https://lab.loman.net/2017/03/09/ultrareads-for-nanopore/).
- *Human training:* A sample of reads from chr1 and chr2 of native R9.4.1 reads (except flowcell FAB49164) from the human reference standard CEPH1463 from nanopore whole human genome sequencing project [Jain et al., 2018].

For testing, the following data sets were used:

- *Klebsiella*: a benchmark set of native R9.4 *K. pneumoniae* reads [Wick et al., 2019]. We only used reads before the sequencing restart.
- *Human testing:* a sample of native R9.4.1 reads from chr14, chr15, chr16 (flowcell FAB49164) from human reference standard CEPH1463 from nanopore whole human genome sequencing project [Jain et al., 2018]

The basic characteristics of all data sets are shown in Table S2.

**Data Set**	**# reads**	**Total length (in bp)**	**Mean read length (in bp)**	**Median read length (in bp)**
Taiyaki set	5000	28.8 Mbp	5769	5459
E. coli training set	2804	90.6 Mbp	32319	21758
Human training set	1323	11.5 Mbp	8705	7111
Human testing set	305	2.7 Mbp	8936	6231
Klebsiella	1788	40.8 Mbp	22613	17547

Table S2: Overview of training and testing sets used in the study.

S3 Training procedure details

We wrote our training pipeline in TensorFlow and the code is available at `https://github.com/fmfi-compbio/coral-training`. Our training schedule uses 6000 miniepochs, one miniepoch being 15 optimizer steps on batch of 100 sequences of length 5004 (5004 being the nearest multiple of 9 targeting our desired sequence length 5000). We use Adam optimizer with a schedule that uses rather high learning rate decaying linearly $LR(t) = 0.01 * (1 - t)$ updated after each miniepoch, where $0 \leq t \leq 1$ denotes progress. We have a short ramp-up period over first 10 miniepochs where we increase learning rate from 0 to the maximum value to avoid "shocking" the model with high learning rate from the start. At the end of training we use standard TensorFlow post-training quantization to convert the model into a format compatible with Edge TPU compiler.

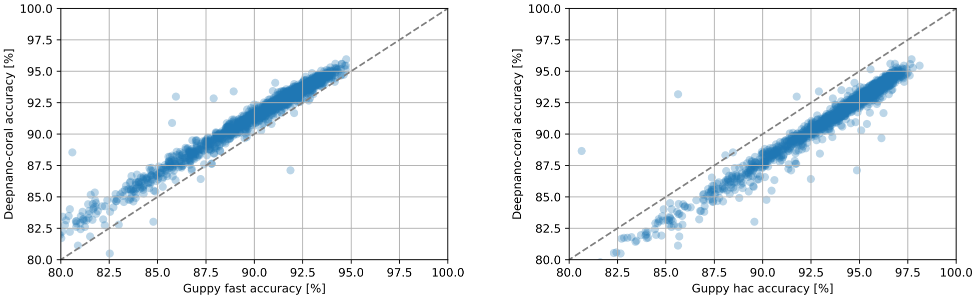

Figure S1: Single-read accuracy comparison between Guppy and DeepNano-Coral. Each dot represents a single sequencing read.

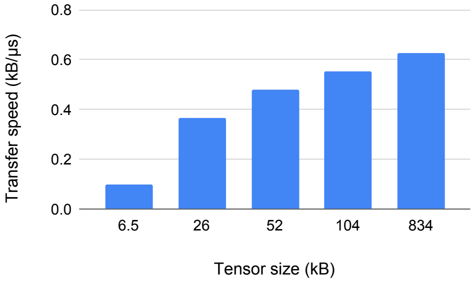

Figure S2: Estimated I/O transfer speed for copying tensor of shape $(4, 1668, C)$ (with C ranging from 1 to 128) to and from the Coral device.

S4 Additional evaluation

Figure S1 shows a comparison between the accuracy of Guppy base calls and DeepNano-Coral on individual sequencing reads. With the exception of a few outliers, there is a strong correlation between accuracy of different base callers, pointing to differences in underlying quality of the signal. Improvement in the accuracy compared to Guppy-fast is consistent for most of the reads.

We benchmarked Coral I/O operation overhead on tensors of varying size. Figure S2 suggests that transfer speed is limited by an overhead for small tensors (e.g. $(4, 1668, 1-4)$) and plateaus at around 0.6kB per microsecond. This is substantially slower than subsequent tensor operations performed on the device.

References

[Jain et al., 2018] Jain, M., Koren, S., Miga, K. H., Quick, J., Rand, A. C., Sasani, T. A., Tyson, J. R., Beggs, A. D., Dilthey, A. T., Fiddes, I. T., et al. (2018). Nanopore sequencing and assembly of a human genome with ultra-long reads. *Nature Biotechnology*, 36(4):338–345.

[Ramachandran et al., 2018] Ramachandran, P., Zoph, B., and Le, Q. V. (2018). Searching for activation functions. In *6th International Conference on Learning Representations (ICLR)*. OpenReview.net.

[Wick et al., 2019] Wick, R. R., Judd, L. M., and Holt, K. E. (2019). Performance of neural network basecalling tools for Oxford Nanopore sequencing. *Genome Biology*, 20(1):129.
